# Effects of ICI 182780 on estrogen receptor expression, fluid absorption and sperm motility in the epididymis of the bonnet monkey

**DOI:** 10.1186/1477-7827-3-10

**Published:** 2005-03-02

**Authors:** Deshpande Shayu, Chenna CS Kesava, Rama Soundarajan, A Jagannadha Rao

**Affiliations:** 1 Department of Biochemistry, Indian Institute of Science, Bangalore- 560012, India; 2 Department of Medicine, Division of Nephrology, University of California San Francisco, SanFrancisco 94143, USA; 3 Department of Clinical Research, University of Bern, 120 Tiefenaustrasse 3004, Bern, Switzerland

## Abstract

**Background:**

The importance of estrogen in regulation of fluid absorption and sperm maturation in the rodent epididymis has been established from studies on estrogen receptor-alpha knockout mice. However, functional studies on the role of estrogen in primate epididymis have been few. The main objective of this study was therefore to extend these observations and systematically analyze the presence and function of estrogen receptors in modulating the function of the primate epididymis, using the bonnet monkey (Macaca radiata) as a model system.

**Methods:**

A steroidal estrogen receptor (ER) antagonist, ICI 182780 (ICI), was administered to adult male bonnet monkeys via mini-osmotic pumps for a duration of 30 to 180 days. The expression of key estrogen-regulated genes (ER-alpha, Na-K ATPase alpha-1 and Aquaporin-1) was examined at specific time points. Further, the effect of ICI in modulating fluid reabsorption in efferent ductules was monitored, and critical sperm-maturation parameters were also analyzed.

**Results:**

Our studies in the bonnet monkey revealed that both ER-alpha and ER-beta were expressed in all the three regions of the epididymis. We observed an increase in ER-alpha mRNA and protein in the caput of ICI-treated monkeys. Steady state mRNA levels of the water-channel protein, Aquaporin-1, was significantly lower in the caput of ICI-treated monkeys compared to controls, whereas the mRNA levels of Na-K ATPase alpha-1 remained unchanged. In vitro incubation of efferent ductules with ICI resulted in two-fold increase in tubular diameter, indicating affected fluid reabsorption capacity. Furthermore, sperm from ICI-treated monkeys were immotile.

**Conclusion:**

Taken together, our results point to an integral role for estrogen in modulating the functions of the bonnet monkey epididymis. This study also demonstrates possible differences in the epididymal physiology of rodents and non-human primates, and thus underscores the significance of reports such as these, that examine the physiology of non-human primates (as opposed to rodents), in an attempt to understand similar events in the human.

## Background

Mammalian testicular spermatozoa are incapable of fertilizing ova. The metamorphosis of immature spermatozoa into mature, functional units capable of progressive motility and fertility, is thought to be the result of a highly regulated and complex series of events that occurs during their transit through the efferent ductules and the epididymis. The epididymis consists of tubules which form a conduit for spermatozoa traversing from the efferent ductules to the vas deferens. It is anatomically divided into three parts- the caput (the head), a narrow central portion- the corpus (the body) and the cauda (the tail). The epithelium lining of these tubules secretes ions and proteins, reabsorbs testicular fluid and creates a specialized luminal environment for the maturation of testicular spermatozoa [1]. These important functions of the efferent ductules and the epididymis are regulated by a complex interplay of growth factors and hormones.

Recent studies point to the involvement of the steroid hormone estrogen, in the regulation of fluid reabsorption in the efferent ductules [2,3]. The action of estrogen is classically mediated via estrogen receptors α and -β (ERα and ERβ), which are present in the male reproductive tract of several species [4]. ERα and ERβ knockouts (αERKO and βERKO) in mice have provided valuable insights into the role of estrogen in male reproductive physiology. The male αERKO mice were infertile [5,6] with gross morphological changes, disrupted spermatogenesis, and dilated efferent ductules due to increased fluid accumulation [7]. In contrast, the male βERKO mice were fertile and had a reproductive tract that appeared normal [8], whereas the male double (α and β) knockout mice were infertile, and their reproductive tract had a αERKO-like morphology. Collectively, these observations suggested that, at least in rodents, functional ERα was necessary to maintain fertility and normal morphology of the efferent ductules. In support of this view, recent studies in mice have shown that estrogen might regulate the expression of key molecules involved in ion transport, resulting in modulation of fluid reabsorption in the efferent ductules [3].

The role of estrogen in the epididymis is, however, less clear. While a high level of ERα expression has been found in the efferent ductules of humans and non-human primates, the expression of ERα in the epididymis of the same species has been sporadic [9]. The expression of ERβ, however, has been detected throughout the male reproductive tract [9]. Now, it is well established that sperm maturation is not intrinsic to sperm cells themselves, but instead requires the interaction of spermatozoa with proteins that are synthesized and secreted by the epididymal epithelium in a highly regionalized manner. Given the potential importance of estrogen during development of the male reproductive system, and the growing possibility that the regional expression of each gene in the epididymis may actually reflect functional differences, the significance of analyzing region-restricted expression patterns of estrogen receptors is obvious. Such a detailed analysis of ERα and ERβ expression pattern, all along the epididymis, will provide valuable information in the quest to determine region-specific functions necessary for sperm maturation. Also, comparative elucidation of the ERα and ERβ expression profiles in the different epididymal segments is a crucial step toward uncovering the regulatory and functional differences (if any) between them.

Studies, in rodents have suggested a possible role for estrogen in modulating epididymal function [10]. Now, although the general organization and functions of the non-human primate epididymis are similar to that of other mammals such as rodents [11], there are obvious limitations in extrapolating results from these models to the primate epididymis. A few studies performed using macaque and baboon tissues have suggested the presence of specific receptors for estrogen in the primate epididymis [12,13]. However, detailed functional studies of primate epididymal expression patterns coupled with mechanistic correlations at the molecular level, have been sparse. Hence, with a view to better understand the role of estrogen in the epididymis of the non-human primate, we initiated studies using the bonnet monkey (*Macaca radiata*) as a model system. Because of its close similarity to the human system, this study using bonnet monkey epididymis, permits meaningful extrapolation to humans. This is all the more important, considering the fact that it is extremely difficult to obtain human epididymal samples (at a reproductively suitable age) for research purpose.

We analyzed the distribution of ERα and ERβ, in all three regions of the bonnet monkey epididymis, using Reverse-Transcription-Polymerase-Chain-Reaction (RT-PCR), Western blot analyses and immunohistochemistry. We next studied the effect of estrogen blockade on the expression pattern of key proteins known to be involved in fluid reabsorption. Estrogen action was blocked using the well-established estrogen receptor antagonist, ICI 182780 (ICI), which can bind both ERα and ERβ. It is important to note that this compound does not cross the blood-brain barrier [14,15] and hence, the levels of gonadotropins and testosterone remain unchanged during this treatment [16]. This allows us to examine authentic estrogen effects. Bonnet monkeys were implanted with mini-osmotic pumps containing ICI for varied durations, the longest being for a period of 180 days, which is greater than the duration of three rounds of spermatogenesis in the monkey [17,18]. The effect of the treatment was studied at the molecular level by analyzing the expression of ERα, Aquaporin-1 (AQP-1) and Na^+^-K^+ ^ATPase-α1, genes that are known to play a key role in modulating fluid reabsorption. It is important to note that among the different regions of the epididymis, the caput is most active in protein synthesis and secretion [19]. Many of the reproductive and somatic genes studied, are either exclusively expressed or exhibit the highest level of expression in this region. Moreover, the caput region of the epididymis has been demonstrated to be extremely sensitive to estrogen action [20,21]. Considering the above facts, our studies were restricted to analysis of the caput region.

Interestingly, in addition to the impaired fluid reabsorption observed in αERKO mice, Eddy et al, noticed that majority of the epididymal sperms collected, showed decreased motility and that even the small fraction of sperms with normal motility were unable to fertilize wild-type oocytes [6], indicating, for the first time, that estrogen was probably essential for sperm function *per se*, in rodents. Our own earlier studies [22] in which Tamoxifen (another Estrogen Receptor antagonist) was chronically administered via Alzet pumps to adult male bonnet monkeys, revealed that these monkeys were infertile and had a very high percentage of abnormal sperms, which exhibited poor motility, although there was no adverse effect on serum testosterone levels. These results suggested the possibility that estrogen may have a role in sperm maturation in non-human primates also.

In an attempt to evaluate the role of estrogen in the regulation of sperm maturation during their transit down the epididymis, we monitored sperm count in all the ICI-treated bonnet monkeys, upto 180 days. Also, in the 180-day ICI treated monkeys, motility of the sperms was analyzed. In this connection, it is pertinent to note that sperm motility is often used as a crucial parameter to assess sperm maturation [23].

## Materials and Methods

### Animals and treatment

Adult male bonnet monkeys (*Macaca radiata*; weighing 6–8 kg) of proven fertility, that exhibited a clear nocturnal surge of serum testosterone, were recruited into the study. The significance of nocturnal surge of serum testosterone as an index of male reproductive function has been described earlier [24,25]. All animals chosen for the study, exhibited sperm counts in the range of 150–500 million/ejaculate. The maintenance of animals has been described previously [26]. All procedures involving use of animals were approved by the Ethics Committee of Indian Institute of Science (Protocol No. 21).

Animals (3 per group) were administered estrogen receptor antagonist ICI 182780 (a pure anti-estrogen; a kind gift from Dr. A. E. Wakeling, Zeneca Pharmaceuticals, U.K.) in propylene glycol (250 μg ICI/day/animal) via Alzet pumps (model 2ML4; Alza Corporation, Palo Alto, CA), which were changed every 28 days. As a control, one set of animals was vehicle (propylene glycol)-treated. At the end of the treatment period (i.e. 30 or 60 days), the animals were subjected to unilateral castration under aseptic conditions, using standard surgical procedures. The epididymal tissue was dissected free of fat, separated into caput, corpus and cauda regions, washed extensively in PBS (pH 7.4) to remove sperms, and processed for immunohistochemistry and RT-PCR analyses. The separation of distinct epididymal regions was performed as follows: regions of one cm from the anterior and posterior ends were dissected out as caput and cauda respectively. One cm region at the center of the epididymis, approximately equidistant from the caput and cauda was delineated as corpus. The tissues were stored in Bouin's fluid for immunohistochemistry or flash frozen in liquid nitrogen and stored at -70°C until required for subsequent RNA extraction. Analysis of sperm motility was carried out with ejaculates of 180-day ICI treated bonnet monkeys.

### Hormone Measurement

Radioimmunoassay methods were based on the procedure standardized in the laboratory [25]. The antiserum to testosterone was raised in rabbits against testosterone 3-carboxy methyloxime BSA conjugate and the cross reactivities with other related steroids were found to be negligible. The intra and inter assay variation was 4.8% and 7.6% respectively. Values reported are uncorrected for recovery.

### Semi-quantitative RT-PCR analysis

Total RNA from epididymal tissue was isolated using TRI reagent (Sigma Chemicals Co., St. Louis, MO) according to manufacturer's instructions. The integrity of the isolated RNA was checked on 1% MOPS-HCHO agarose gel and the quantity of RNA estimated spectrophotometrically. Reverse transcription was carried out as described earlier [27,28] with appropriate controls.

cDNA amplifications used highly specific forward and reverse primers with an initial heating at 94°C for 3 minutes, followed by 28 cycles of 94°C for 1 minute, annealing temperature for 1 minute and 72°C for 1 minute, on a PCR Thermal Cycler (MJ Research Inc., USA). PCR details are compiled in Table-1. An aliquot of 10 μl of the PCR product was electrophoresed on a 1.5% agarose gel and visualized upon staining with ethidium bromide. Each figure is representative of at least 4 independent experiments. Amplification of GAPDH (Glyceraldehyde 3-phosphate dehydrogenase) served as internal control.

**Table 1:** List of primers and conditions employed for PCR

Gene	Primer Sequence	Annealing Temp (°C)	Product size (base pairs)
ERα	FP 5' gag aca tga gag ctg cca ac 3' RP 5' cca aga gca agt tag gag ca 3'	55	381
AQP-1	FP 5' cag cat ctt ccg tgc cct cat gta 3' RP 5' cat act cct cca cct ggc cgc tgg 3'	58	495
Na^+^-K^+ ^ATPase-α1	FP 5' aaa ctt agc ctt gat gag ctt 3' RP 5' tcc atg atc ttt gaa ctt tta 3'	55	344
GAPDH	FP 5' gga gtc aac gga ttt ggt 3' RP 5' gtg atg gga ttt cca ttg at 3'	54	206

FP: Forward Primer

RP: Reverse Primer

The authenticity of all the RTPCR products described in this study was confirmed by sequencing, following their purification from low-melting agarose gels using the commercially available GFX™ PCR DNA/Gel Band Purification kit (Amersham Pharmacia Biotech., UK). Sequencing was performed at the DNA sequencing facility, IISc, using the ABI Prism 377 automated DNA sequencer.

### Preparation of protein lysates

Tissue samples were washed extensively with PBS (pH 7.4) and homogenized in the presence of ice-cold lysis buffer (50 mM Tris pH 8.0,150 mM Sodium Chloride, 0.1% SDS, 0.02% Sodium Azide, 1% Nonidet-P40 and 0.5% Sodium Deoxycholate) containing 100 μg/ml PMSF and 1 μg/ml Aprotinin. Protein lysates were clarified by centrifugation at 15000 × g for 20 minutes at 4°C, and the protein content was determined by the Bradford's assay [29]. Aliquots of protein lysate were stored at -70°C until analyzed. All the chemicals used for lysis were purchased from Sigma Chemical Co., St. Louis, Mo.

### Western Blot analyses

Equal quantities of protein (100 μg) were electrophoresed on 10% SDS-polyacrylamide gels and transferred onto nitrocellulose (Sartorius AG, Germany) membranes, using a semi-dry transfer apparatus. Western blot analysis was performed as described earlier [27]. Following hybridization, the blot was stripped and re-probed (using the protocol recommended by the manufacturers, Amersham Pharmacia Biotech, UK) for actin which served as the internal control for assessing equality of protein-loading. ERα antibody was used at a dilution of 1:200. ERβ and actin primary antibodies were used at a dilution of 1:400. All antibodies were purchased from Santa Cruz Biotechnology Inc., CA. Each figure is representative of at least 3 independent experiments.

### Immunohistochemistry

Monkey epididymal tissue was fixed in Bouin's fluid, dehydrated, embedded in paraffin using standard procedures [30] and sectioned at 8 μm thickness. Following deparaffinization and rehydration, the sections were pre-treated with methanol-hydrogen peroxide to quench endogenous peroxidase activity, followed by a microwave treatment in 0.01 M (pH 6.0) citrate buffer [31]. The sections were stained according to the double PAP (peroxidase-anti-peroxidase) technique [32] with diaminobenzidine (Sigma Chemicals Co., St. Louis, MO, USA) as the chromogen. The sections were then counter-stained with toluidine blue, dehydrated with graded concentrations of ethanol and xylene and analyzed by microscopy. Goat anti-rabbit IgG serum and rabbit anti-goat IgG serum, adsorbed against human proteins was obtained from Antibodies Inc., Davis, CA. The rabbit and goat peroxidase-anti-peroxidase complexes were obtained from Jackson ImmunoResearch laboratories, West Grove PA and diaminobenzidine was obtained from Aldrich, Inc., Milwaukee, WI. Anti-ERα and -ERβ antibodies, purchased from SantaCruz Biotechnology Inc., CA, were used at an optimum dilution of 1:200.

As a positive control for ERα immuno-peroxidase staining, we used mouse uterus tissue [33]. As a positive control for ERβ staining we used rat ventral prostate tissue [34]. Pre-incubation of the ERα antibody with the corresponding blocking peptide (SantaCruz Biotechnology Inc., CA) prior to staining, served as an appropriate negative control. As a negative control for absence of ERβ staining, we used rat liver tissue [34]. Omission of the primary antibody from the experiment served as another negative control.

### Analyzing the effect of ICI on tubular diameter of efferent ductules

Following castration of untreated monkeys, the efferent ductules were identified and separated out by micro-dissection, minced and placed in the medium of the composition given below, for 24 hours. Both the ends were ligated with sterile 4-0 cat gut (Johnson, India), to prevent the in-flow of medium. The ligated efferent ductules were incubated in M-199 medium (Sigma Chemical Co., St. Louis, MO) containing Bovine Serum Albumin (500 μg/ml; Sigma Chemical Co., St. Louis, MO), 17β-estradiol (1 nM; Steraloids Inc., Wilton, NH, USA) and dihydrotestosterone (1 nM; Steraloids Inc., Wilton, NH, USA), at 34°C, in humidified 95% air/5% CO2, for 24 hours, with (test) or without (control) ICI (1 μM). At the end of the total incubation period, the samples were rapidly fixed in Bouin's and subsequently embedded in paraffin using standard procedures [30]. 5 μm sections were cut, stained in hematoxylin and eosin, and analyzed by light microscopy. The diameters of both control and ICI-treated efferent tubules were measured at the minimum point and tabulated.

### Sperm count and motility analysis

The details of electroejaculation and sperm analysis have been described earlier [35]. Semen from the monkeys was collected by electroejaculation by the penile method [35]. Ejaculates were kept at 37°C throughout the period of examination, which did not exceed a total of 90 minutes. Sperm count was determined using Neubauer hemacytometer following 1:200 dilution of the semen in saline. The number of live spermatozoa was determined using eosin-nigrosin stain. Motility of the semen was analyzed using a computer-assisted semen analysis system (CASA, Hamilton-Thorne Research, MA). Semen sample was diluted 20 times with Tris buffer pH 7.4 and employed for analysis. One drop of the diluted semen sample was added to the Makler chamber (10 μM) and inserted into the feeding chamber of CASA. The sperm from each monkey were analyzed for the following motion parameters: percentage of motile sperm, percentage of progressively motile sperm, average path velocity, curvilinear velocity, lateral head displacement, straightness ([straight line velocity/average path velocity] × 100) and linearity ([straight line velocity/curvilinear velocity] × 100). The analysis was carried out with the following instrument settings: 30 frames at a rate of 60 Hz; minimum cell size, 5 pixels; minimum contrast, 80. All these procedures were performed at 37°C.

### Statistical analysis of data (where applicable)

Data are represented as mean ± S.E. of at least three independent experiments performed with the same treatment protocol. For comparison among groups involving two columns significance was evaluated using the unpaired two-tailed *t*-test. For comparison of data involving three columns, statistical significance was obtained using the Kruskal-Wallis ANOVA followed by Neuman-Keuls test. In both instances a *P *value less than or equal to 0.05 was considered to be statistically significant.

## Results

### 1. RT-PCR analysis for ERα, AQP-1 and Na^+^-K^+ ^ATPase-α1 in the three regions of the epididymis

**Figure 1:**
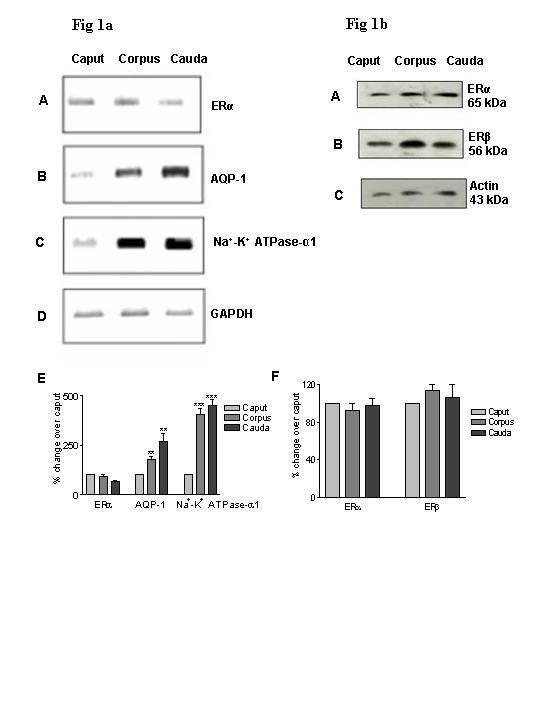
**a RT-PCR analyses of ERα (Panel A), AQP-1 (Panel B), Na^+^-K^+ ^ATPase-α1 (Panel C) and GAPDH (Panel D) in the bonnet monkey epididymis: **RNA isolated from the caput, corpus and cauda regions of the bonnet monkey epididymis was reverse transcribed and the cDNA so obtained was subjected to semi-quantitative PCR in the linear range of amplification with GAPDH amplification as an internal control. **Panel E **is a graphical representation of this data wherein, the densitometric signal intensities obtained for ERα, AQP-1, Na^+^-K^+ ^ATPase-α1 were normalized against that of GAPDH and plotted as percent change of the individual intensities with respect to the caput region. Data represents Mean ± SEM of three independent experiments. **P < 0.05, ***P < 0.001. **b Western blot analyses for ERα and ERβ in the bonnet monkey epididymis: **100 μg each of protein lysates obtained from caput, corpus and cauda regions of the bonnet monkey epididymis was electrophoresed on 10% SDS-PAGE, transferred onto a nitrocellulose membrane, and probed with antibody specific to ERα. The blot was stripped and reprobed with an antibody specific to ERβ. Following this, the blot was once again stripped with an antibody specific to actin which served as an internal control for assessing equality of protein loading. **Panel F **is a graphical representation of this data wherein, the densitometric signal intensities obtained for ERα and ERβ were normalized against that of actin and plotted as percent change of the individual intensities with respect to the caput region. The figure is representative of aleast three independent experiments.

In order to ascertain the presence of ERα in the bonnet monkey epididymis, RNA from the caput, corpus and cauda was subjected to RT-PCR analysis using specific primers. All PCRs were performed within the linear range of amplification with GAPDH as the internal control. High level of ERα message was detected in the caput, corpus and cauda regions (Fig 1a). The three regions did not differ appreciably in their level of expression. It has been well documented that the efferent ductules of rodents express components like AQP-1 and Na^+^-K^+ ^ATPase, which are important for fluid absorption [3]. Accordingly we analyzed whether these components of the fluid absorption machinery were present in the bonnet monkey epididymis and further, their distribution within the three regions. Analysis of the steady-state mRNA levels of these genes revealed specific signals for each of these components in all the three regions of the monkey epididymis, with varying levels of expression. We observed a two-fold higher expression of AQP-1 in the cauda compared to the caput. The level of expression of Na^+^-K^+ ^ATPase-α1 was four-fold higher in the corpus and cauda compared to the caput.

#### Western blot analyses for ERα and ERβ in the three regions of the bonnet monkey epididymis

We observed specific signals for ERα (65 kDa) and ERβ (a prominent band of 56 kDa) in the caput, corpus and cauda regions (Fig 1b). Comparative analysis revealed no significant difference in the intensity of expression of the two receptor subtypes (at the protein level) in the three regions of the monkey epididymis.

### 2. Immunolocalization of ERα and ERβ in the three regions of the bonnet monkey epididymis

**Figure 2:**
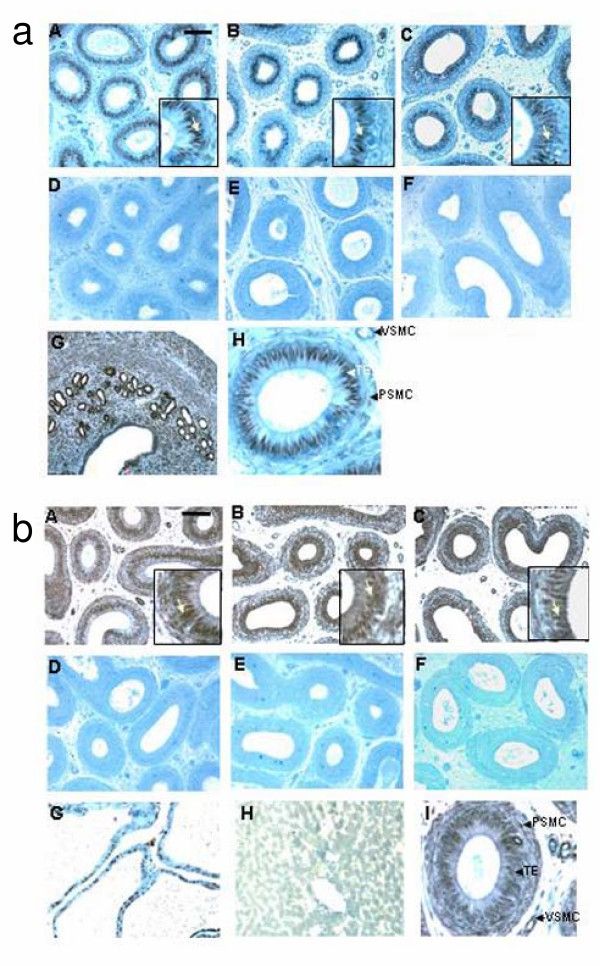
**Immunohistochemical localization of ERα and ERβ in the bonnet monkey epididymis: **a. ERα staining: Immuno-peroxidase staining for ERα showing intense nuclear staining in caput (**Panel A**), corpus (**Panel B**) and cauda (**Panel C**) regions of the bonnet monkey epididymis. Staining was most intense in the tubular epithelial cells lining the lumen (inset; staining indicated by arrow). **Panels D-F **show the negative controls for each region, wherein the primary antibody was incubated with the blocking peptide. **Panel G **shows intense staining in the nuclei of mouse uterus tissue, which served as the positive control. **Panel H **shows an enlarged epididymal tubule representing the staining pattern in different cell types- while staining was intense in the nuclei of the tubular epithelium, weak staining was observed in the smooth muscle cells surrounding the tubule and that of the vascular ducts ; TE-tubular epithelial cell, PSMC- peri-tubular smooth muscle cell, VSMC- vascular smooth muscle cell. Bar in Panel A = 100 μ b. The caput, corpus and cauda regions of the bonnet monkey epididymis (**Panels A-C**, respectively) show intense staining for ERβ both in the nuclei of the epithelial cells lining the lumen (inset; staining indicated by arrow), and the surrounding stroma. **Panels D-F **represent the negative controls for each region wherein the addition of the primary antibody was omitted. **Panel G **shows intense staining in rat ventral prostate tissue, which served as a positive control. **Panel H **shows absence of ERβ staining in rat liver tissue, which was used as a negative control. **Panel I **shows an enlarged epididymal tubule depicting staining in the various cell types, in and around the tubule- considerable staining was also observed in the smooth muscle cells; TE-tubular epithelial cell, PSMC-peri-tubular smooth muscle cell, VSMC- vascular smooth muscle cell. Bar in Panel A = 100 μ.

Intense staining for ERα was observed in all the three regions i.e. the caput, corpus and cauda, of the bonnet monkey epididymis (Fig 2a). Staining was localized mainly to the nuclei of the epithelial cells lining the lumen. However, weak staining was also observed in the surrounding peri-tubular smooth muscle nuclei and in the vascular smooth muscle nuclei. Pre-incubation of the primary antibody with the blocking peptide completely abolished staining establishing the specificity of the antibody. An intense signal was also observed with mouse uterus which was used as a positive control.

The three regions of the epididymis stained intensely for ERβ (Fig 2b). Intense staining was observed in the peri-tubular and vascular smooth muscle nuclei. Staining was also observed in the tubular epithelial cell nuclei of caput, corpus and cauda regions. The cytoplasm in these cells was also weakly stained. This staining is in sharp contrast to the ERα staining which was mostly restricted to the tubular epithelial cell nuclei. As a positive control for ERβ staining, we used rat ventral prostate tissue, in which intense nuclear staining could be observed. We did not observe any staining with rat liver tissue, which was used as negative control.

### 3. Effect of ICI treatment on the bonnet monkey serum testosterone levels

**Figure 3:**
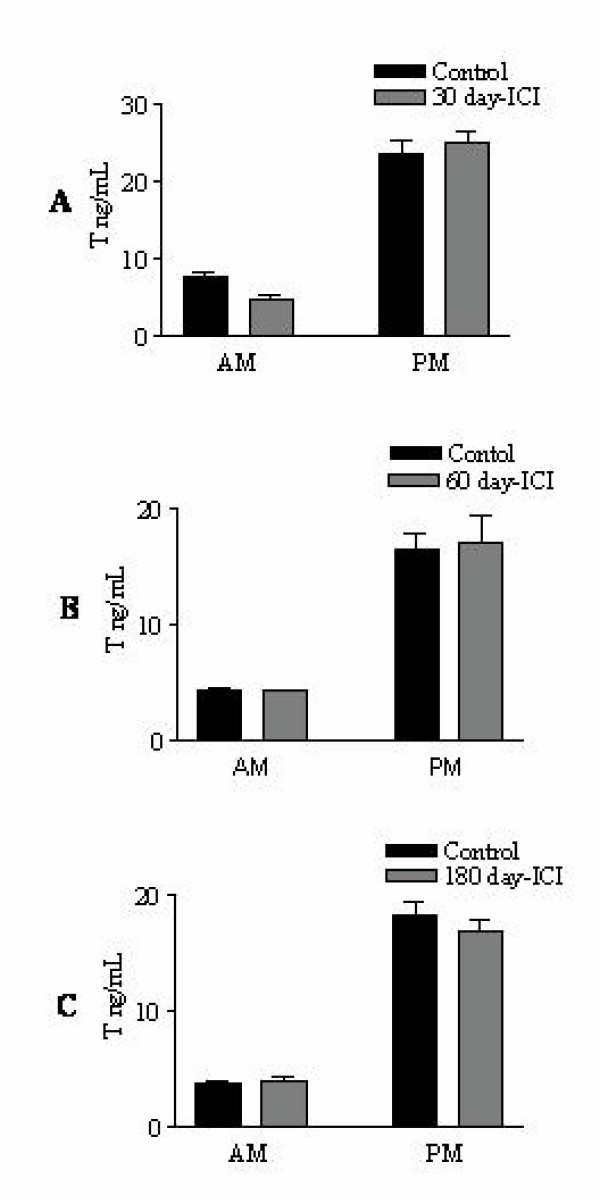
**Serum testosterone levels in the ICI-treated adult male bonnet monkeys: **Following vehicle or ICI treatment in monkeys for 30, 60 or 180 days, serum testosterone levels were analyzed by radio-immuno assay. We did not observe any significant changes in serum testosterone levels. Nocturnal surge of testosterone (PM values compared to AM values) also remained unchanged following ICI treatment. Data shown is a Mean ± SEM of three experiments.

Our results showed that there was no change in serum testosterone levels in the 30-day, 60-day or 180-day ICI-treated monkeys (Fig 3). Nocturnal surge of serum testosterone also remained unaltered. This is in accordance with previous reports that ICI does not cross the blood-brain barrier [15] and hence does not alter the hormonal profile.

### 4. Expression profile of ERα, AQP-1 and Na^+^-K^+ ^ATPase-α1 in the caput region of 30-day and 60-day ICI treated monkey: RT-PCR analysis

**Figure 4:**
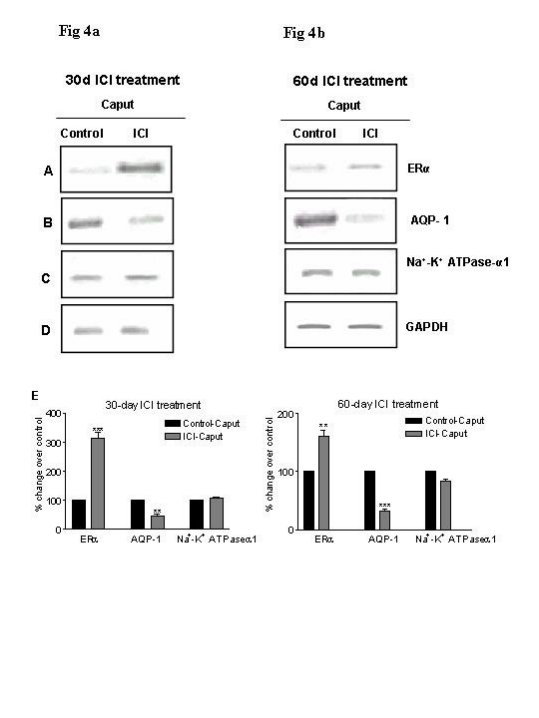
**a-b RT-PCR analysis for ERα, AQP-1, Na^+^-K^+ ^ATPase-α1 and GAPDH in the caput regions of control and 30-day (Fig 4a) and 60-day (Fig 4b) ICI treated monkeys: **RNA isolated from the caput region of vehicle-and ICI-treated bonnet monkeys, was reverse transcribed and the cDNA was subjected to semi-quantitative PCR in the linear range of amplification with GAPDH amplification as an internal control. The intensity of ERα expression (**Panel A**) increased upon ICI treatment compared to that of vehicle-treated controls, with the increase being appreciably greater in the 30-day treatment period. AQP-1 levels were reduced in the ICI-treated group compared to controls (**Panel B**). There was no discernable change in the Na^+^-K^+ ^ATPase-α1 levels between the ICI-treated groups and vehicle controls (**Panel C**). **Panel E **is a graphical representation of this data wherein, the densitometric signal intensities obtained for ERα, AQP-1 and Na-K ATPAse-α1 were normalized to that of GAPDH (**Panel D**). Data represents Mean ± SEM of three experiments. **P < 0.05, ***P < 0.001.

In order to assess the effect of ICI treatment on the steady state mRNA levels of ERα, AQP-1 and Na^+^-K^+ ^ATPase-α1, RNA from the vehicle and ICI treated-caput regions was subjected to RTPCR analyses using specific primers. A significant 4-fold increase was found for ERα in the 30-day ICI-treated samples compared to the vehicle control (Fig 4a). We also observed a significant increase in the ERα message in the 60-day ICI-treated samples (Fig 4b). A two-fold reduction in the message for AQP-1 was observed in the caput of both 30- and 60-day ICI treated monkeys compared to the corresponding vehicle controls. In sharp contrast, we did not observe any significant changes in the expression of Na^+^-K^+ ^ATPase-α1 between the vehicle control and ICI-treated samples (Fig 4a,b). These results indicated that ICI treatment significantly affects ERα and AQP-1 mRNA levels in the caput region of the epididymis.

### 5. ERα expression in the caput region of vehicle and ICI-treated bonnet monkey epididymis: Immunohistochemistry

**Figure 5:**
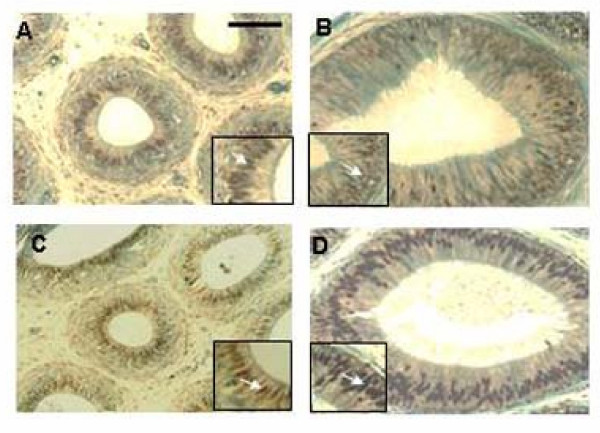
**Expression of ERα in the caput region of 30- and 60-day ICI-treated bonnet monkeys: **Immunoperoxidase staining for ERα showed striking differences in expression intensities following 30 and 60 days of ICI treatment. Staining as seen previously was nuclear (inset; indicated by arrow). Compared to the 30-day vehicle-treated control caput sample (**Panel A**), the intensity of ERα staining was unchanged in the caput after 30-day ICI treatment (**Panel B**). Staining was very intense in the 60-day ICI-treated caput (**Panel D**) in comparison to the corresponding 60-day vehicle treated control (**Panel C**). Bar in Panel A = 100 μ.

To ascertain whether the increase in the ERα mRNA observed in the ICI-treated caput samples was actually reflected at the protein level, we immunolocalized ERα in these sections using a well characterized antibody (Fig 5). Interestingly, no change was appreciable in the ERα signal in the 30-day ICI-treated caput samples (compared to vehicle control), although we had observed a significant increase in the ERα mRNA at this time-point (compare with Fig 4). However, a striking increase in the ERα expression was seen in the 60-day ICI-treated caput samples (compared to the corresponding vehicle control), reflecting the increase in the mRNA levels observed in Fig 4.

### 6. Effect of ICI on the fluid reabsorption capacity of efferent ductules

**Figure 6:**
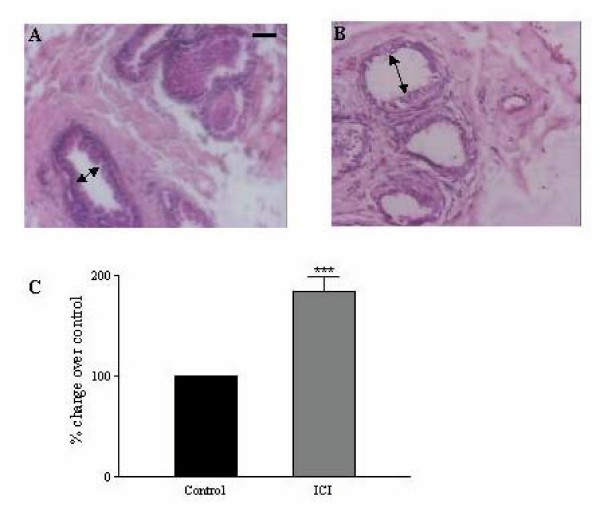
**Change in the luminal diameter of efferent ductules after *in-vitro *incubation with ICI: **Efferent ductules were minced and tissue fragments of 2–3 mm were incubated in M199 medium containing dihydrotestosterone (1 nM), 17β-estradiol (1 nM) with or without ICI (1 μM) for 24 hr. The ends of the tubules were ligated with a sterile catgut and incubated in a fresh medium of the same composition, for further 24 hr. Subsequent to this total incubation period of 48 hr, the ductules were rapidly fixed in Bouin's fluid and embedded in paraffin. Sections were stained in hematoxylin and eosin and diameter of the tubules measured. In absence of ICI, the control (**Panel A**) shows reduced luminal diameter compared to ICI-incubated efferent ductules (**Panel B**). **Panel C **is a graphical representation of the luminal diameters. Values represent Mean ± SEM from three independent experiments. Bar in Panel A = 100 μ; *** *P *< 0.001.

We determined whether *in-vitro *incubation of efferent ductules with ICI affected fluid reabsorption. Efferent ductules were chosen for the study, as previous reports have shown that these absorb the majority of the testicular fluid and hence we reasoned that the effect of ICI on fluid reabsorption should be easily discernible in these tubules. Following 48 hr of the total incubation duration with ICI, we observed an almost two-fold increase in the efferent ductule diameter compared to control ductules incubated without ICI (Fig 6). These results support the observations (in rodents) made by other workers, who showed that efferent ductules are very sensitive to lack of estrogen [36]. Since this experiment was carried out with efferent ductules from normal monkeys, it ruled out any possible involvement of testicular factors being modulated by *in-vivo *ICI treatment and indirectly regulating fluid absorption in efferent ductules.

### 7. Effect of ICI on sperm count and motility

**Figure 7:**
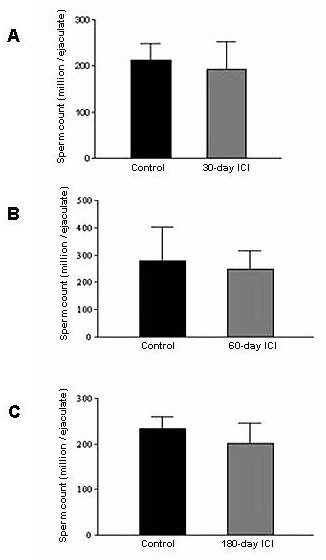
**Effect of ICI treatment on the sperm count in bonnet monkeys: **Semen from the monkeys from vehicle- and ICI-treated bonnet monkeys was collected by electro-ejaculation, and sperm count was determined using Neubauer hemacytometer following 1:200 dilution of the semen in saline. As seen from the **Panels A-C**, sperm count does not change considerably in the ICI-treated groups compared to the control. Data represented is a Mean ± SEM of three independent experiments.

Short-term (30-day period) and long-term (180-day period) ICI treatment had no adverse effect on the sperm count in all the monkeys tested at the given dose and duration of treatment (Fig 7). All the samples analyzed had more than 90% viable sperms as tested by eosin-nigrosin stain (data not shown).

#### Effect of ICI on sperm motility parameters: CASA (compiled in Table 2)

**Table 2:** Effect of ICI on sperm motility parameters by Computer-Assisted Semen Analysis (CASA)

**Particulars**	**Control**		**Experimental**	
	I	II	I	II

Motile (%)	100	100	9	7
Progressive (%)	100	75	0	0
Path velocity (μm/s)	53.2	48.8	26.4	34
Progressive velocity (μm/s)	34.2	36.1	24.7	27.9
Track speed (μm/s)	101.7	85.1	33.3	41.2
Lateral amplitude (μm)	10.2	8.0	0	0
Beat frequency (Hz)	24.8	19.9	0	0
Straightness (%)	63	64	94	76
Linearity (%)	37	46	74	76

I and II represent individual samples

For this experiment, since only a small group of monkeys could be analyzed, the data could not be statistically evaluated. Nevertheless, it is pertinent to note that in both the 180-day ICI-treated monkeys tested, a drastic reduction in progressive motility was observed. A decrease in the beat frequency and lateral amplitude of the sperms was also observed in the same animals.

## Discussion

To our knowledge, this is the first study demonstrating the presence of both the estrogen receptors, ERα and -β, in the three regions of the bonnet monkey epididymis. In contrast to previous studies [9,37], which were unable to detect ERα in the marmoset monkey epididymis, our results show the unequivocal expression of ERα at both the mRNA and protein level in all three regions of the bonnet monkey epididymis. Localization of ERα protein was prominent in the nuclei of the tubular epithelial cells lining the lumen while ERβ was expressed abundantly both in the epithelium and the stroma. The physiological significance of this differential staining pattern of the two receptor subtypes in *Macaca radiata *epididymis is at present, unclear. The presence of ERβ in the stromal cells, in addition to the epithelium, is interesting as stromal-epithelial cell interactions have been shown to modulate the response to estrogen [38]. Although there is considerable regional overlap in the expression of both ER-α and -β, further specific studies are needed to assess the relative abundance and co-expression of the two receptors. Nevertheless, the presence of both the receptors indicates a plausible role for estrogen in the physiology of the bonnet monkey epididymis.

ER antagonists provide a good tool to study the effect of lack of estrogen action on the target tissue. ICI 182780, an ER antagonist, when administered to mice for 35 days produced an α ERKO-effect [39]. In the current study, the effect of ICI treatment on the caput region of the bonnet monkey epididymis was examined. ERα mRNA expression was increased in the caput region of both 30- and 60-day ICI-treated monkeys. An appreciable increase in ERα protein expression was also noted in the caput of 60-day ICI-treated group. At this juncture, it is important to note that, most studies in rodents have actually reported a decrease in the receptor level with ICI treatment [40-42], with the exception of the endometrium where no change in the receptor level was seen with ICI treatment[43]. Interestingly, results from our lab also showed decreased ERα expression in the caput of ICI-treated rodents (unpublished data). It is therefore possible that the increased expression of the receptor in the ICI-treated caput of the bonnet monkey could be a species-specific effect. This difference underscores the significance of studies such as these, that examine the physiology of non-human primates (as opposed to rodents), in an attempt to understand similar events in the human.

Impaired sperm production in male αERKO mice has been suggested to be due to disruption of estrogen action within the somatic cells of the testis and the excurrent duct system wherein fluid absorption fails to occur. Fluid transport is predominantly facilitated by a family of water channel proteins called aquaporins. This movement of water across the cell membrane is dependent on the concentration gradient, which is maintained by ion exchangers like the Na^+^-K^+^-ATPase pump, Na^+^-H^+ ^exchangers, chloride channels, etc. Studies in rodent efferent ductules suggested that estrogen may be involved in the regulation of aquaporin-1 (AQP-1) and Na^+^-K^+ ^ATPase-α1 expression [39,44], although the precise mechanism is still unclear.

Our experiments in the bonnet monkey revealed robust expression of AQP-1 and Na^+^-K^+^-ATPase-α1 in all the three regions of the epididymis. This is in accordance with previous studies in rodent efferent ductules that also showed high expression of AQP-1 and Na^+^-K^+^-ATPase-α1, two proteins important for maintenance of fluid and ion balance [2]. The effect of ICI treatment on the expression of these proteins was similar for both the 30-day and 60-day treatments. A significant decrease was observed in AQP-1 mRNA levels with ICI treatment, while Na^+^-K^+^-ATPase-α1 levels did not vary appreciably. These results are consistent with earlier findings, which showed that AQP-1 expression was modulated by estrogen in the efferent ductules of both rodents and monkeys [44,45]. In view of all these observations, it is surprising that there was apparently no adverse effect on the efferent ductules in AQP1-knockout mice [44]. As suggested, this could be attributed to the existence of compensatory mechanisms like paracellular movement of water or involvement of other isoforms of AQP.

Additionally, our results showed that incubation of efferent ductules with ICI *in vitro*, led to an increase in lumen diameter indicating absence of involvement of testicular factors in regulating fluid absorption. Although the increase in ERα expression upon ICI treatment was in sharp contrast to the observations made earlier, the downstream effects of ICI treatment, i.e., decease in AQP-1 expression and increase in lumen diameter, were similar to those observed in the rodent model. Taken together, these results indicate that fluid absorption in the bonnet monkey excurrent ducts is affected by ICI treatment, suggesting a role for estrogen in this process.

As a final read-out of ICI-treatment, we analyzed critical sperm-maturation parameters in the ICI-treated monkeys. Studies have shown that administration of aromatase-inhibitors reduced the transit time of spermatozoa and that such spermatozoa were not capable of fertilization [46,47]. Sperm from the αERKO mouse also showed decreased motility [6]. In our study too, we observed that sperm from the 180-day ICI-treated monkeys, revealed a precipitous drop in motility. The treatment affected the beat-frequency and progressive motility of sperms, which is indicative of a loss in their fertilization potential. Interestingly, these treatment-induced effects occurred in the absence of any obvious alteration in sperm morphology or sperm production, as sperm count in the treated monkeys was normal. This indicated that the treatment was probably affecting the maturation process and not so much the production process. It is known that sperm proteins and the membrane undergo several modifications during maturation in the epididymis. It is therefore reasonable to predict that some of the processes involved in maturation may be altered, leading to decreased sperm motility. These effects were observed in the absence of any detectable change in testosterone levels, and hence it is reasonable to conclude that the observed defects in motility could be due to blockade of estrogen action. In support of this view, a recent report suggested that aromatase expression in human spermatozoa was important for sperm motility [48]. Understanding the role of ER-α and -β in fluid reabsorption and sperm motility, is thus of high physiological relevance for normal fertility. Further studies are required to assess if the observed reduction in sperm motility also results in decreased fertility. Future studies are aimed at identifying candidate sperm maturation factors that are subject to estrogen regulation in the bonnet monkey epididymis.

## Conclusion

In conclusion, this study, for the first time, provides evidence for the presence of estrogen receptors in the bonnet monkey epididymis. The results obtained so far clearly establish that the bonnet monkey epididymis is also a target for estrogen action and that several genes reported to be involved in the regulation of fluid absorption in the rodents, are also regulated by estrogen in the monkey epididymis. We show that ICI-treatment alters epididymal and sperm-maturation functions. This study also demonstrates possible differences in the epididymal physiology of rodents and non-human primates, and thus underscores the significance of reports such as these, that examine the physiology of non-human primates (as opposed to rodents), in an attempt to understand similar events in the human.

## List of abbreviations used

**ER**, Estrogen Receptor; **ERα**, Estrogen Receptor-α; **ERβ**, Estrogen Receptor-β; **αERKO**, α-Estrogen Receptor Knockout mouse; **βERKO**, β-Estrogen Receptor Knockout mouse; **Na^+^-K^+ ^ATPase-α1**, Sodium-Potassium-ATPase α1-subunit; **AQP-1**, Aquaporin-1; **RTPCR**, Reverse-Transcription-Polymerase-Chain-Reaction; **GAPDH**, Glyceraldehyde-3-Phosphate-Dehydrogenase; **CASA**, Computer-Assisted Semen Analysis

## Authors' contributions

SD carried out the hormone measurement and sperm maturation assays, performed the statistical analyses, and drafted the manuscript. CCS performed the RTPCR and Western blot analyses. RS participated in the design of the study, performed the immunohistochemical analyses and helped to draft the manuscript. AJR conceived the study, participated in its design and co-ordination, and helped to draft the manuscript. All authors read and approved the manuscript.
